# The Role of Vitamins in Neurodegenerative Disease: An Update

**DOI:** 10.3390/biomedicines9101284

**Published:** 2021-09-22

**Authors:** Sachchida Nand Rai, Payal Singh, Harry W.M. Steinbusch, Emanuel Vamanu, Ghulam Ashraf, Mohan Prasad Singh

**Affiliations:** 1 Centre of Biotechnology, University of Allahabad, Prayagraj 211002, India; raibiochem@gmail.com; 2 Department of Zoology, MMV, Banaras Hindu University, Varanasi 221005, India; payalsingh200012@gmail.com; 3 Department of Cellular Neuroscience, Faculty of Health, Medicine & Life Sciences, Maastricht University, 6211 LK Maastricht, The Netherlands; h.steinbusch@maastrichtuniversity.nl; 4 Department of Cognitive Neuroscience, DGIST, Daegu 42988, Korea; 5 Faculty of Biotechnology, The University of Agronomic Science and Veterinary Medicine, 59 Marasti blvd, 1 District, 011464 Bucharest, Romania; 6 Pre-Clinical Research Unit, King Fahd Medical Research Center, King Abdulaziz University, Jeddah 21589, Saudi Arabia; ashraf.gm@gmail.com; 7 Department of Medical Laboratory Technology, Faculty of Applied Medical Sciences, King Abdulaziz University, Jeddah 21589, Saudi Arabia

**Keywords:** vitamins, neurodegenerative disease, Parkinson’s disease, Alzheimer’s disease, Huntington disease, Prion disease

## Abstract

Acquiring the recommended daily allowance of vitamins is crucial for maintaining homeostatic balance in humans and other animals. A deficiency in or dysregulation of vitamins adversely affects the neuronal metabolism, which may lead to neurodegenerative diseases. In this article, we discuss how novel vitamin-based approaches aid in attenuating abnormal neuronal functioning in neurodegeneration-based brain diseases such as Alzheimer’s disease, Parkinson’s disease, Huntington’s disease, Amyotrophic lateral sclerosis, and Prion disease. Vitamins show their therapeutic activity in Parkinson’s disease by antioxidative and anti-inflammatory activity. In addition, different water- and lipid-soluble vitamins have also prevented amyloid beta and tau pathology. On the other hand, some results also show no correlation between vitamin action and the prevention of neurodegenerative diseases. Some vitamins also exhibit toxic activity too. This review discusses both the beneficial and null effects of vitamin supplementation for neurological disorders. The detailed mechanism of action of both water- and lipid-soluble vitamins is addressed in the manuscript. Hormesis is also an essential factor that is very helpful to determine the effective dose of vitamins. PubMed, Google Scholar, Web of Science, and Scopus were employed to conduct the literature search of original articles, review articles, and meta-analyses.

## 1. Introduction

In infants and elderly people, vitamin deficiency is common [1]. Prolonged deficiencies in vitamins lead to malnutrition and severe health issues [2,3]. Interestingly, the typical balanced diet of a healthy population has a rich number of vitamins, which prevent several diseases [4,5]. This has prompted researchers to explore the role of different vitamins in the development and progression of diseases. For this review, we focus on vitamins relative to neurodegenerative diseases.

In general, vitamins are considered organic compounds that are required for the development and normal functioning of the body. The body cannot synthesize vitamins, either at all or not in sufficient quantities. As such, they must be obtained through the diet. Importantly, vitamins commonly function as antioxidants or enzymatic cofactors [6,7]. There are two main categories of vitamins: fat-soluble vitamins, which are stored in the body until your body needs them, and water-soluble vitamins, which are not stored in the body, so a continuous exogenous daily supply is required [8,9].

Generally, vitamins offer a significant advantage for neurodegenerative diseases. Both water- and lipid-soluble vitamins prevent Parkinson’s and Alzheimer’s disease in a substantial manner. The α-synuclein toxicity has been prevented by vitamin supplementation. In addition, vitamins also exert their protective effects on the dopamine transporter. Amyloid and tau pathologies are also progressively prevented by a higher dose of vitamins. Vitamins also show their therapeutic properties for Huntington’s, Prion, and multiple sclerosis diseases. However, some studies show the contrasting activity of vitamins too for the diseases mentioned above. Some vitamins also show toxicity too. Therefore, there is a solid need for the role of vitamins in neurodegenerative diseases to be described clearly. The role of different vitamins and their detailed mechanisms of action are included in their respective sections.

## 2. Water-Soluble Vitamins

Water-soluble vitamins (WSVs) are structurally and functionally distinct compounds vital for normal cellular functions, growth, and development. The WSV is generally considered as a micronutrient, and the deficiency of which causes severe diseases such as neurological diseases, growth retardation, or intestinal diseases [10,11]. The essential WSVs are vitamin B1 (Thiamine), vitamin B2 (Riboflavin), vitamin B3 (Niacin, Nicotinic Acid), vitamin B5 (Pantothenic Acid), vitamin B6 (Pyridoxine and derivatives), vitamin B7 (Biotin, Vitamin H), vitamin B9 (Folate, Folacin), vitamin B12 (Cobalamin), and vitamin C (Ascorbate, Dehydroascorbate). Thiamine is also known as the Anti-beriberi factor and Aneurin. The molecular weight of thiamine is 265.36 g/mol. The reaction between ATP and thiamine forms active coenzyme thiamine pyrophosphate. Thiamine pyrophosphate plays a very important role in carbohydrate metabolism. Thiamine is a colorless compound with a chemical formula C_12_H_17_N_4_OS. It is insoluble in the organic solvent while soluble in polar solvents such as water. For the proper maintenance of the heart, nervous system, and digestive system, thiamine plays a crucial role [12]. The molecular formula of riboflavin (Vitamin B2) is C17H20N4O6, and the molecular weight is 376.4 g/mol. Riboflavin is the precursor of two vital coenzymes, flavin mononucleotide (FMN) and flavin adenine dinucleotide (FAD). FAD and FMN are involved in the oxidation/reduction reaction. These coenzymes also take part in the metabolism of proteins, carbohydrates, and lipids. For healthy hair, skin, and nails, riboflavin is very important because it can also modulate the antioxidant enzyme glutathione reductase [13]. The chemical formula of nicotinic acid (Vitamin B3) is C6H5NO2 or C5H4NCOOH, and its molecular weight is 123.11 g/mol. It is also called niacin, pyridine-3-carboxylic acid, and 3-pyridinecarboxylic acid. It is essential for reducing the bad cholesterol (LDL) and enhancing the good cholesterol (HDL) in our body. That is why it is used to manage dyslipidemia. The level of serum aminotransferases might elevate because niacin and its high dose might be responsible for acute liver injury. The tase of vitamin B3 is feebly acidic, and it is an odorless crystalline powder. Niacin is used as a vasodilator agent, antilipemic drug, and also an antidote [14]. The chemical formula and molecular weight of pantothenic acid (Vitamin B5) are C_9_H_17_NO_5_ and 219.23 g/mol, respectively. Pantothenic acid exhibits strong antioxidative properties and is widely found in both animal and plant tissues. This vitamin is the main part of the Vitamin B2 complex and coenzyme A. It is involved in the metabolism of lipid, carbohydrates, and proteins. This vitamin also takes part in the synthesis of hormones, various neurotransmitters, hemoglobin, and lipids [15]. The molecular weight and chemical formula of pyridoxine (Vitamin B6) are 169.18 g/mol and C_18_H_11_NO_3_. It is also called Pyridoxol and Gravidox. Pyridoxine is converted into pyridoxal phosphate (PLP) that plays a significant contribution in the transamination reaction. PLP also helps in the synthesis of essential neurotransmitters such as serotonin and norepinephrine. Pyridoxine is involved in the metabolism of glycogen and amino acids [16]. Biotin (Vitamin B7) is also called Vitamin H, and C_10_H_16_N_2_O_3_S and 244.31 g/mol are the chemical formula and molecular weight of this enzyme. This is involved in the carboxylation reaction. This enzyme is required for proper growth and metabolism [17]. Folate (Vitamin B9) is also considered as folic acid and vitamin M. The chemical formula and molecular weight of this enzyme are C_19_H_19_N_7_O_6_ and 441.4 g/mol, respectively. This enzyme plays a role in the metabolism of amino acids and carbon transfer reactions. Moreover, folate is utilized in the production of red blood cells and hematopoiesis [18]. Cobalamin (Vitamin B12) is also called cyanocobalamin. Its molecular weight and chemical formula are 1355.4 g/mol and C_63_H_88_CoN_14_O_14_P, respectively. It is a cobalt-containing coordination compound synthesized by intestinal microbes and requires intrinsic factors for absorption through the intestine. Its deficiency caused megaloblastic anemia and pernicious anemia. In addition, several neurological lesions have been observed due to the lack of this enzyme [19]. Vitamin C or ascorbic acid is one of the important antioxidants taken in the diet. The molecular weight and chemical formula of this enzyme are 176.12 g/mol and C_6_H_8_O_6_ or HC_6_H_7_O_6_. It is found in lemons, oranges, and other citrus fruits. This enzyme is essential in the diet and cannot be produced by humans. It is involved in the synthesis of collagen. Its deficiency causes several complications such as scurvy. Its overdose is also responsible for jaundice and acute liver injury [20]. WSVs mainly function as cofactors for an associated enzyme and ultimately modulate the biological activity of specific enzymes [21,22].

## 3. Fat-Soluble Vitamins

As the name implies, fat-soluble vitamins (FSVs) dissolve in fats and oils. FSVs, namely A, D, E, and K, are absorbed in the intestine [8,23]. Vitamin A is also called retinol or all-trans-Retinol. The molecular weight and chemical formula of this vitamin are 286.5 g/mol and C_20_H_30_O, respectively. This vitamin is required for the normal functioning of the eyes, and it is also involved in the modulation of immune function. Reproductive organ functioning is also mediated by this enzyme. Totals of 300 to 700 μg for children and 700 to 900 μg for adults are recommended in the diet. A higher dose might cause several complications such as cirrhosis and portal hypertension, etc. [24]. Vitamin D3 (cholecalciferol) is the active form of Vitamin D. The chemical formula and molecular weight of vitamin D3 are C_27_H_44_O and 384.6 g/mol, respectively. It is produced in the skin by ultraviolet light and also is obtained in the diet and belongs to the steroid hormones category. This vitamin regulates the level of calcium and phosphorus in the body by bone mineralization and demineralization. It also affects gene expression by binding on the receptor of vitamin D [25]. Vitamin E is also considered as alpha-Tocopherol and 5,7,8-Trimethyltocol. The molecular weight and chemical formula of this enzyme are 430.7 g/mol and C_29_H_50_O_2,_ respectively. It shows potent cytoprotective and vital antioxidant properties. This vitamin protects our body from harmful oxidative damage. It maintains the permeability of the cell membrane by neutralizing the reactive oxygen species. It is also involved in the regulation of reproductive functions [26]. Vitamin K is also considered as Kinadion, Konakion, Mephyton, Monodion. The chemical formula and molecular weights are C_31_H_46_O_2_ and 450.7 g/mol. This enzyme is required for the normal blood clotting reaction. Multiple forms of this vitamin are known as Vitamin K2 (menaquinone), Vitamin K1 (phytomenadione), and Vitamin K3 (menadione). Butter, egg yolk, green leafy vegetables, cheese, and liver are good sources of this vitamin [27]. Clinically, the deficiency in FSV is described as night blindness (vitamin A), osteomalacia (vitamin D), increased oxidative cell stress (vitamin E), and hemorrhage (vitamin K). In the last few decades, additional potential actions for FSVs have been suggested, such as vitamin A and D deficiencies being indirectly linked to cancer, diabetes mellitus, and several immune disorders [28,29,30,31,32]. The FSVs, vitamin A and vitamin D, mainly act through nuclear receptors to control the expression of different genes [21,33]. Vitamin E is a powerful antioxidant, and vitamin K plays a key role in blood clotting. As research progresses, additional roles and mechanisms of action of FSVs will be further described.

In this review, we will discuss the role of WSVs and FSVs in the progression of neurodegenerative diseases. In general, vitamins play a neuroprotective role; several derivatives of vitamins have been tested to treat various neurological diseases and have produced significant findings [34,35,36,37,38,39]. Therefore, in the following sections, we will reveal the role of different vitamins one by one in the context of specific neurodegenerative diseases.

## 4. Vitamins in Parkinson’s Disease

Parkinson’s disease (PD), characterized by several motor and non-motor symptoms, arises due to the degeneration of dopaminergic neurons in the midbrain region [40,41,42,43,44]. This disease has a high incidence in the aging population [45]. Effective treatments, which can stop the initiation or prevent the progression of PD, are not currently available. Only therapies to alleviate the symptoms are available. However, symptoms become worse following prolonged treatment [46]. Now, research is focused on identifying different compounds that can prevent the progression and initiation of PD [47]. Accordingly, in recent years, a role for several vitamins has been suggested for the treatment of PD [48,49]. These vitamin treatments may offer fewer if any side effects and may improve on currently available therapies for PD.

**Figure 1:**
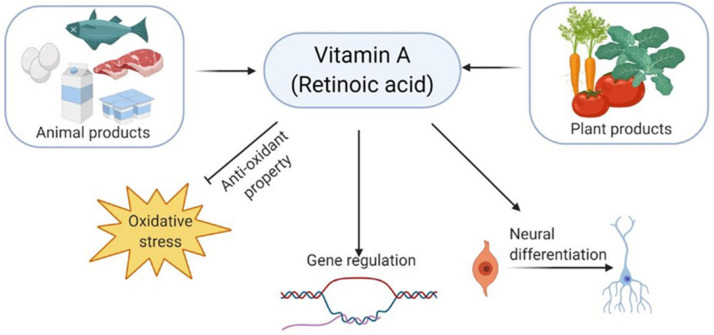
Animal and plant sources of vitamin A (Retinoic acid). Vitamin A shows potent antioxidant activity. Gene regulation and neural differentiation are affected by vitamin A supplementation.

One of the major causative factors responsible for the progression of PD is oxidative stress, and vitamin A (VitA), along with its derivatives such as retinoic acid (RA), exhibits strong antioxidative activity [30,50]. VitA is obtained from both animal and plant sources [51]. Fish, meat, and dairy products are animal sources of preformed VitA (retinol and retinyl ester) (Figure 1) [51]. On the other hand, plant sources provide the precursors of VitA in the form of carotenoids (β-carotene, α-carotene, and β-cryptoxanthin) [51,52]. VitA is involved in multiple signaling pathways that regulate gene expression [30,53]. Specifically, in the central nervous system (CNS), VitA regulates several vital processes such as controlling neural cell differentiation and patterning in neural tube formation [54,55].

### 4.1. Vitamins Based Clinical Studies in Parkinson’s Disease

One study demonstrated high concentrations of VitA and its derivatives in the human post-mortem frontal lobe cortex [56]. This is a biomarker-based clinical study that assessed the therapeutic impact of VitA in the frontal lobe cortex. The frontal lobe cortex showed an age-related decline in retinol and its derivatives compared to the occipital cortex. Further studies will be needed to explore and compare other brain areas for a similar type of activity. Additionally, a Singaporean Chinese cohort-based study also suggested no correlation between dietary antioxidants, such as carotenoids and vitamins (vitamin A, C, and E), and risk of developing PD [57]. A comparative study suggested the relation between risk of PD and intake of carotenoids, VitE and VitC. This is a follow-up study on both male and female PD patients. A total of 47.331 men and 76.890 women were followed up to 12 and 14 years, respectively. Food frequency questionnaires on these PD patients were also noted in this study. None of these vitamins and even multivitamins were scientifically associated with the risk of PD in these patients. On the other hand, a food-derived high intake of VitE leads to a reduced risk of PD in both male and female patients. The risk of PD also gets reduced after the consumption of nuts. A multivitamin study showed that PD risk is not significantly reduced after intake of vitamins E and C, carotenoids. A multivitamin approach rich in vitamin E showed a better therapeutic effect than carotenoids and vitamin C. Therefore, this study suggested that dietary food rich in VitE reduces the risk for PD compared to carotenoids or VitC. Thus, VitE supplementation is protective for PD [58]. Therefore, a broader level study is needed regarding the therapeutic potential of VitA, VitC, VitE, and its derivatives in PD.

In many neurodegenerative diseases including PD, increased homocysteine levels, a sulphur-containing metabolite of methionine biosynthesis, were found to have multiple neurotoxic effects [59]. As such, in PD patients with enhanced homocysteine levels, there was a strong correlation between homocysteine level and PD pathogenesis [60,61,62]. Enhanced levels of homocysteine were responsible for the death of nigrostriatal dopaminergic neurons in PD patients. Thus, regulating the level of homocysteine may prevent PD progression [63,64]. Vitamin B (VitB) acts as a cofactor in the synthesis of methionine from homocysteine, and researchers have suggested that the level of homocysteine was strongly correlated with VitB levels [22,65]. Supplementary VitB has been shown to reduce the level of homocysteine in blood plasma [66,67]. Regardless, limited studies regarding the roles of the different types of VitB in PD pathologies were performed. Of interest, Vitamin B12 (VitB12) levels were decreased in PD patients as compared to normal healthy controls. In addition, a reduced risk of PD was found in those who consumed sufficient amounts of dietary Vitamin B6 (VitB6) [68,69]. No significant change was observed regarding folate (VitB9) concentration in PD patients versus normal healthy controls [69,70].

Interestingly, most research on VitB and homocysteine in brain function has mainly focused on three of the eight B-vitamins (VitB9, VitB12, VitB6), and, thus far, the results have been equivocal [22,66]. However, it has been suggested that treatments utilizing a combination of all might yield better results because of the inter-related cellular functions of the eight VitB types [71]. Thiamine deficiency causes death of the dopaminergic neurons in PD. Supplementation of thiamin was responsible for the delay in the progression and death of dopaminergic neurons in PD [72]. An open level study suggested that Parkinsonian symptoms become reversed significantly by the intramuscular administration of a high dose of thiamine to PD patients without any side effects [73].

Of additional interest, PD patients with olfactory dysfunction observed 2–8 years before the display of symptoms demonstrated low dietary thiamine (VitB1) and folate (VitB9) density [74,75]. Thus, at the early stage of PD, thiamine and folate levels effectively regulate the olfactory system and might serve as an important screening tool for detecting PD risk. A case report shows that bradykinesia and rigidity were significantly improved by niacin treatment for those with rashes in the skin and unacceptable nightmares [76]. In conclusion, because of the limited number of studies on the various types of VitB for treatments and/or the diagnosis of PD, more research is needed before advocating their usage in PD therapies.

**Figure 2:**
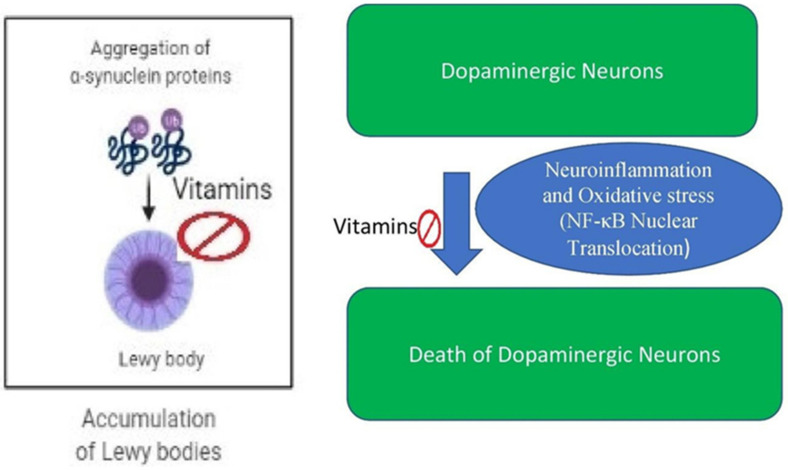
Vitamins prevent the oligomerization of alpha-synuclein into Lewy bodies. Neuroinflammation and oxidative stress are mainly responsible for the death of dopaminergic neurons via the NF-κB pathway. Vitamins ultimately prevent the death of dopaminergic neurons by inhibiting the neuroinflammation and oxidative stress in Parkinson’s disease. Vitamins prevent the nuclear translocation of NF-κB and associated activation of proinflammatory cytokines.

Humans show a dose-response towards VitC as high levels can be toxic [77,78], but when VitC is maintained at homeostatic concentrations, there is the positive effect of reducing oxidative stress, one of the leading causes of neurodegenerative diseases (Figure 2) [79].

A clinical trial shows that ascorbic acid (200 mg) is very effective for elderly PD patients. In this clinical trial, the authors have suggested that ascorbic acid improves the absorption of levodopa (100 mg levodopa and 10 mg carbidopa) in a significant manner in PD patients. Therefore, the combinatorial therapy of ascorbic acid with levodopa might get a better result than treatment of one alone [80]. However, there is still controversy in the potential use of VitC for the treatment of PD, and further studies are desired [21].

Researchers have also suggested a link between vitamin D (VitD) and PD, and despite all efforts, it is not clear yet if a deficiency in VitD is responsible for PD or a consequence of PD [81]. Cutaneous levels of VitD are directly correlated to sunlight exposure [82,83], and ultraviolet exposure enhances the level of VitD in our body [84,85]. Bone density is directly associated with the concentration of VitD and consequently postural stability [86]; however, the role of VitD in the brain is less understood. Calcitriol (1,25- dihydroxy vitamin D3) is an active metabolite and the most studied form of VitD [87]. Compared to age-matched healthy controls, PD patients have a much lower concentration of calcitriol in their blood plasma, which may be related to bone health and fracture risk [88,89]. Interestingly, the receptor of calcitriol is also expressed in the CNS [90]. Thus, VitD mediates its effects in PD through its active form, calcitriol, and associated receptors present in the CNS [86,91]. One study shows an inverse association and toxic activity of vitamin D in PD [92]. Another study also indicates the toxicity of VitD as responsible for reversible symptoms of Parkinsonism [93].

Regrettably, a recent meta-analysis found few studies focusing on vitamin D supplementation in PD treatments. This study shows an inverse relationship between VitD level and risk and severity of PD in 2866 PD patients [94]. There is a limited response of VitD supplementation (≥400 IU/day) in early PD patients, and this warrants further study to justify its therapeutic potential [95]. Therefore, more cohort studies of early and late PD patients are needed to illuminate the connection between VitD and PD further.

Vitamin E (VitE) is a well-known antioxidant found in vegetables, and it has multiple therapeutic uses [96]. From the mid-1990s, the role of VitE has been demonstrated in several neurological diseases including PD [97]. Similar to other vitamins, VitE exhibits a strong connection with PD [97]. Results from a recent study demonstrated that the effect of VitE was age- and sex-independent and showed an inverse relation between VitE intake and PD occurrence [97]. Consequently, diets rich in VitE may minimize the risk associated with PD [98,99].

The progression of PD might be controlled by high-dose alpha-tocopherol and ascorbate as shown by a pilot study. Further clinical trials at a broader level will be needed to confirm the protective efficacy of alpha-tocopherol and ascorbate [100]. As shown by a clinical trial, high dose vitamin E (2000 IU vitamin E orally per day) treatment enhanced its concentration in the cerebrospinal fluid (CSF) of an early untreated PD patient. High CSF and a high brain concentration of alpha-tocopherol show a protective effect on the PD patients [101]. One study also shows that there is not any relation between serum VitE and risk of PD [102]. Another study recommended daily multivitamin supplementation that should contain at least 30 IU of alpha-tocopherol instead of 400 IU of alpha-tocopherol in the affected individuals. This combination showed an improved therapeutic response in PD patients [103]. A multivitamin approach by using vitamin E, vitamin C, and carotenoids is not beneficial in reducing PD risk. Instead, dietary vitamin supplementation rich in VitE shows enhanced therapeutic potential to reduce the PD risk. A very high dose might lead to losses in the therapeutic activity of Vitamin E [104]. Therefore, care should be taken, and a broader level of clinical study is needed to prove the therapeutic potential of VitE. A case report on an old PD patient shows the enhanced therapeutic activity of multivitamin-multimineral supplementation with *Ginkgo biloba* [105]. A large population-based study is necessary to confirm the same.

### 4.2. Vitamin Based Animal Studies in Parkinson’s Disease

The cellular retinol-binding protein found on the blood-brain barrier (BBB) allows the brain easy access to VitA and its derivatives [106]. In the CNS, the prominent target of RA action is the nigrostriatal dopaminergic system [107,108]. RA-receptor heterodimers bind to the RA-response element within the promoter region and drive the transcription of the dopamine 2 receptor gene. As such, RA receptor knockout mice exhibited reduced expression of the D2 dopamine receptors [60]. Thus, the RA receptor effectively regulates the homeostatic regulation of the nigrostriatal dopaminergic system as supported by multiple pieces of evidence [108,109,110,111]. Therefore, further investigation into the incorporation of VitA in prospective PD therapies may prove fruitful. A recent study suggested that there is no effect of oral supplementation of retinol in the 6-hydroxydopamine intoxicated Wistar rat model [112]. RA receptor based therapeutic approaches might be beneficial in preventing the progression of PD and suggest the mechanism of action behind it [113]. Lycopene is an important carotenoid that also exhibits its effectiveness in the MPTP-induced Parkinsonian mouse model. The lycopene treatment reversed physiological anomalies, oxidative stress, neurochemical abnormalities, and apoptosis. Lycopene shows anti-apoptotic and antioxidative properties in this PD mouse model [114]. Lycopene also protects the cognitive decline in the rotenone-induced PD model [115].

Vitamin C (VitC), which humans cannot synthesize due to the absence of the enzyme L-gulonolactone oxidase, has many health benefits including essential antioxidant activities [116]. For example, treatments with VitC mitigated the PD-like phenotype of dopaminergic neuron degeneration and locomotor deficits in a UCH-L1 gene knockdown Drosophila PD model (Figure 2) [117]. Recently, a study explored the dose-dependent effects of VitC using this knockdown fly model. The authors suggested that for PD treatment, VitC dose-response activity should not be ignored as high concentrations of VitC had adverse effects on behavior and locomotion [118]. Ascorbic acid (100 mg/kg) administration just before the 20 min of MPTP intoxication showed a protective effect by its antioxidative activity in the BALB/c PD model (Figure 2) [119].

In an animal model of PD, VitD inhibits neuroinflammation by regulating microglial activity and protects the death of dopaminergic neurons (Figure 2) [120]. In a hemiparkinsonian rat model, VitD protects the death of dopaminergic neurons by inhibiting oxidative stress and neuroinflammation [121].

The PD-like symptoms and associated pathology regarding mitochondrial abnormalities and synaptic impairment in a PD knockdown model were significantly improved by VitE supplementation [97]. Chronic intake of VitE (500 mg/kg-diet) lowers the death of dopaminergic neurons in the substantia nigra of the zitter mutant rat model of PD [122]. However, many researchers have shown contrasting findings regarding the dietary intake of VitE for treatment of PD [123]. Tocotrienols (T3s) are well-known members of the VitE family that also offer neuroprotective potential through their antioxidative activity [124]. Biochemical and behavioral evidence have suggested that the PD progression induced by intrastriatal injection 6-OHDA was ameliorated by VitE treatment in the PD rat model [125]. Similarly, behavioral, neurochemical, and biochemical studies proved that VitE benefits the rotenone-induced rat model [126]. Histochemical and biochemical evidence also suggested that the repeated intramuscular administration of vitamin E (24 I.U./kg, i.m) offers the significant neuroprotective property in an early rat PD model induced by unilateral intrastriatal 6-hydroxydopamine (12.5 microg/5 microl) [127]. Alpha-tocopherol also exhibited a similar neuroprotective activity in the unilateral 6-OHDA model and might be used as an effective PD drug [128].

Results from a study demonstrated that a combination of a higher dose of Coenzyme Q10 (CoQ10) (600 mg/kg/day) with levodopa (10 mg/kg/day) significantly protected subjects from neurodegeneration in a rotenone-induced rat model of Parkinsonism as compared to a low dose combination of CoQ10 (200 mg/kg/day) with levodopa (10 mg/kg/day). The higher dose of CoQ10 with levodopa improved abnormalities in the electron transport chain and exhibited anti-apoptotic activity. Striatal dopamine levels were considerably restored by this treatment paradigm [129].

### 4.3. Vitamins in Cell-Based Parkinson’s Disease

A pluripotent stem-cell-based study shows that RA-γ is the most effective among α and β RA receptors in forming striatopallidal-like neurons in PD by affecting the dopamine 2 receptor gene [130]. Niacin (VitB3) shows potent anti-inflammatory activity through its receptor GPR109A by inhibiting the nuclear translocation of NF-κB in a lipopolysaccharide (LPS)-induced RAW264.7 cell model of PD. This nuclear inhibition of NF-κB prevented the upregulation of proinflammatory cytokines and the associated neurodegeneration in this PD model [131]. NADH is the active coenzyme of VitB3. NADH causes enhanced release of dopamine from the striatal slices at a 350 microM concentration in vitro. There is no effect of NADH (10 or 100 mg/kg) in vivo. For the synthesis and regeneration of tetrahydrobiopterin, NADH is needed. In addition, tetrahydrobiopterin is needed to synthesize Tyrosine hydroxylase, a rate-limiting enzyme in the synthesis of dopamine. A study will be required to validate the in vitro findings in an in vivo model [132]. In a cellular model of PD, the survival rate and energy activity were improved significantly by Nicotinamide mononucleotide (NMN). In the human neuroblastoma cell line SK-N-SH, ascorbic acid (200 microM) led to enhanced expression (three-fold) of tyrosine hydroxylase (TH) after 5 days of treatment. Consequently, dopamine synthesis was increased as a result of enhanced expression of TH. Therefore, ascorbic acid might be utilized in early PD as a potential anti-Parkinsonian agent [133]. In the cellular model, ascorbic acid also exhibits its therapeutic potential against levodopa-induced neurotoxicity [134]. MN ameliorated the process of apoptosis in this cellular model [135].

Further, studies have demonstrated that 1,25-dihydroxyvitamin D3 exhibited strong anti-inflammatory and neuroprotective effects, and reduced the neurotoxin-induced microglial activation and expression of proinflammatory cytokines in experimental models of inflammation-mediated neurodegenerative disease (Figure 2) [136,137].

In a cellular PD model, T3s exhibited cytoprotective activity through the estrogen receptor-activated β-PI3K/Akt pathway [124].

In conclusion, numerous cell-, animal-, and human-based studies have found that vitamins have beneficial effects in the prevention and/or treatment of PD due to their antioxidant properties and other biological functions such as regulating gene expression. However, some studies disagree [21]. Specifically, some studies also show that there is no impact regarding the therapeutic potential of vitamins. In addition, the dose is the very crucial factor that decides the efficacy of the action of vitamins in the PD. Therefore, more clinical studies are necessary to clarify the potential use of vitamin supplementation in PD therapeutics.

## 5. Vitamins in Alzheimer’s Disease

Worldwide, Alzheimer’s disease (AD) is ranked first in occurrence among all neurodegenerative diseases [105]. In the aging population, AD is the most common cause of dementia [138]. Clinical symptoms of AD begin with cognitive impairments, which further progress into dementia [139]. AD neuropathology includes the progressive degeneration of neurons, the formation of amyloid (Aβ) plaques, and neurofibrillary tangles (NFT) of the hyperphosphorylated tau protein [140,141]. As such, post-mortem analysis of AD patient brains reveals a reduction in the size of the cerebral cortex and abnormal deposits inside and around neurons [138]. Similar to other neurodegenerative diseases, one of the leading causes responsible for progressive neurodegeneration in AD is oxidative stress [142]. Accumulating evidence has shown that the antioxidative activity of vitamins may be beneficial in the treatment of AD. As such, vitamins have been used as adjuvants in AD therapy [6,143].

### 5.1. Vitamins Based Clinical Studies in Alzheimer’s Disease

In AD treatment, nicotinamide can be used as an adjuvant therapy because it is a potent inhibitor of Poly (ADP-ribose) polymerase 1 [144]. Multiple pieces of evidence have suggested that compared to healthy individuals with intact neurocognitive function, AD patients show reduced levels of VitA, VitB, and VitC in blood serum [145]. Meta-analytic studies have found reduced serum levels of VitA, VitB [6,9,21], VitC, VitD, VitE, and VitK in AD patients [146,147]. Moreover, reduced levels of VitA, VitC, and VitE were observed in dementia patients as compared to healthy controls [148,149]. Interestingly, a cross-sectional and prospective study reported that a combination of VitC and VitE supplements reduced the prevalence and incidence of AD; however, they made no report of VitA, and found no association of VitB intake with AD [150].

Cognitive function in US women was significantly improved by long-term treatment with retinoids [151]. Clinical trials on RA and associated derivatives might confirm its therapeutic efficacy and role as a biomarker in AD patients.

Gut microbiota also might affect retinoid signaling in AD. Dynamic gut microbiota may directly link with retinoic acid to mediate therapeutic responses in AD [152]. In the early AD stage, there is no correlation between Retinol binding protein 4 and AD. Therefore, RBP4 is not used as a clinical biomarker in early AD [153]. Failures of RA signaling and associated RA deficiency are linked with age-related cognitive decline in AD. Thus, RA therapeutic activity is directly linked with AD and associated symptoms [154].

Several types of B vitamins (VitB9, VitB12, VitB6, and VitB2) are involved in the metabolism of homocysteine [155]. Elevated levels of total plasma homocysteine lead to cognitive impairments, which may ultimately lead to dementia [66,156,157]. Several studies have demonstrated that VitB supplementation lowers total homocysteine in the treatment for cognitive decline [158,159,160]. In an unbiased analysis, Douaud et al. showed that AD pathology, characterized by atrophy of cerebral gray matter, was reduced by VitB supplementation, reducing the total homocysteine in serum plasma [156]. In contrast, a meta-analysis on randomized control trials suggests that no improvements in cognitive impairment have been observed in therapies that reduce total homocysteine with VitB supplementation [161,162,163]. As such, results from a 26-week randomized, double-blind, placebo- controlled study of Taiwanese AD patients given a multivitamin supplement containing vitamins B6, B12, and B9 in addition to acetylcholinesterase inhibitor treatment demonstrated a decrease in the concentration of serum homocysteine but no beneficial effects on cognition or the daily living activity of the AD patients [164]. Another clinical study showed that a high dose of B vitamins (VitB9, VitB12, VitB6), while effectively lowering homocysteine levels, did not affect cognition in individuals with mild to moderate AD [165]. However, in older MCI patients, vitamin B prevented cognitive decline as demonstrated by a randomized placebo-controlled trial [166]. On the other hand, there is no effect of a 2-year treatment of vitamin B on the elevated level of homocysteine and cognitive performance as shown by secondary data from a RCT [167]. Higher vitamin B12 and folate exhibited potent therapeutic activity and improved cognitive performance in a cross-sectional study on AD [168]. The screening of presymptomatic AD cannot be diagnosed by measuring the serum folic and vitamin B12 levels as shown by a Turkish three-center based study [169]. Thus, the role of vitamin B and folic acid for MCI and AD is very speculative and needs a complete investigation. Therefore, a complete evaluation of the therapeutic efficacy of vitamin B and folate in MCI and AD on a broader population might solve the puzzle mentioned above. Significant cognitive impairment with progressive dementia is associated with thiamine deficiency. These symptoms have been improved by the supplementation of thiamine in affected individuals [170]. In elderly patients, reversible dementia is associated with a deficiency in cobalamin [171].

**Figure 3:**
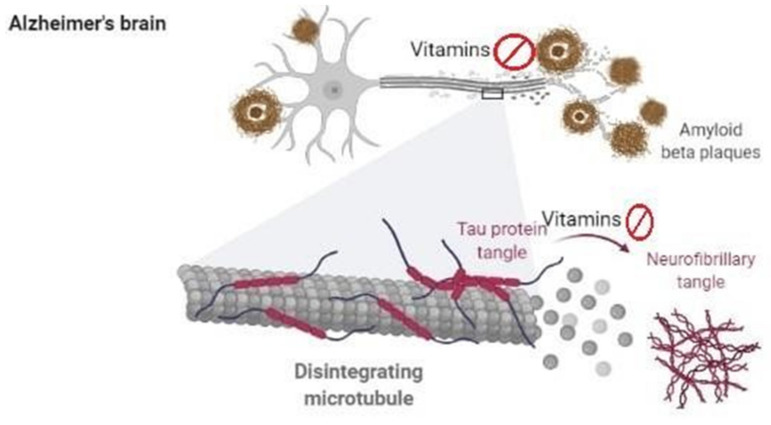
This figure suggests the therapeutic potential of vitamins in Alzheimer’s disease. Vitamins prevent Aβ plaque formation by inhibiting the aggregation of beta-amyloid plaques. Vitamins also prevented the tau protein aggregation and its oligomerization into neurofibrillary tangles.

Vitamin B12 inhibits the tau fibrillization and formation of the neurofibrillary tangle. Thus, VitB12 prevents the tau aggregation and ultimately neurofibrillary tangle formation that might progress the severity of AD (Figure 3) [172]. A Havana, Cuba based study also suggests a relationship between the level of homocysteine and vitamins among older AD patients. In this study, a total of 424 peoples above or equal to the age of 65 were included in which 131 were MCI, 43 AD, and, in 250 individuals, no sign of cognitive impairment was detected. As compared to healthy participants, the level of vitamin A, C, and B2 was reduced significantly among AD patients. In addition, in the same AD patients and MCI patients, the homocysteine level was elevated significantly compared to healthy individuals. The level of vitamin B12, folic acid, and thiamine was unrelated in all groups. The authors concluded that in MCI and AD, various vitamin deficiencies are directly related to impairment in the metabolism of homocysteine [173]. Although this is a very small-scale study, a larger population-based study is needed to find any valid correlation between different B vitamins and hyperhomocysteinemia among AD and MCI patients. In patients with folate deficiency, cognitive impairment was ameliorated by folate supplementation for a short period [174]. Inflammation is one of the major factors that lead to progressive neurodegeneration in AD. A clinical trial suggested that folic acid shows anti-inflammatory solid activity and prevents neurodegeneration in AD. ChiCTR-TRC-13003246 is the registration number of this clinical trial conducted in China [175]. A greater understanding of the relevance of VitB and homocysteine metabolism to cognitive function is wanted.

The risk of AD also increases due to a low serum level of VitD [176,177]. Calcium levels and the parathyroid hormone, along with specific cytokines, regulate the concentration of the active form of VitD, calcitriol. The inactive form of VitD crosses the BBB, and, inside glial and neuronal cells, it is converted into the active form by the enzyme CYP27B1 [178,179]. Microglial cells are also responsible for converting provitamin D into the active form of VitD [180]. Calcitriol controls the synthesis of the nerve growth factor (NGF) and ultimately governs the process of neuronal cell differentiation and maturation. In addition, calcitriol regulates the synthesis of the glial cell-line-derived neurotrophic factor (GDNF) [181,182]. Both the NGF and GDNF regulate learning and memory through the septohippocampal pathway. With advancing age, the level of the NGF decreases [183]. NGF levels are also reduced in AD patients [184]. Amyloid precursor protein (APP) concentration is effectively modulated by the NGF [184]. NGF signaling interruption leads to an up-regulation of APP levels and an increased production of Abeta intracellular aggregates [185]. Of great interest is that the active form of VitD and its analogs induce NGF expression [177,186], which presents a possible mechanism by which VitD could indirectly improve AD pathology.

Cognitive function is affected by the B12 levels as suggested by an elderly Korean population-based study [187].

VitE plays an important role in improving cognitive function along with memory deficits [188]. VitE can be combined with other antioxidants to promote the efficacy of the treatment strategies effectively [189]. Despite several pieces of evidence regarding the antioxidant activity of VitE, the role of VitE is controversial [190].

In contrast, clinical trials regarding the activity of VitE in AD showed conflicting findings [190]. One such trial, reporting the impact of VitE in conjunction with donepezil supplementation for mild cognitively impaired (MCI) patients (early stage of AD), found no added benefit for VitE supplementation [191]. This double-blinded study treated MCI patients with 2000 IU of VitE daily and 10 mg of donepezil daily or a placebo for three years. There was no effect of VitE for MCI; however, donepezil lowered the rate of progression of MCI towards AD for the first 12 months and longer for apolipoprotein E4 carriers [192]. Slower functional decline was observed as a result of 2000 IU/d of alpha-tocopherol in AD patients compared to controls in a collaborative randomized trial-based study [193]. In conclusion, as it is unclear whether prolonged and synergistic treatments with VitE are beneficial for the management of AD, broader population studies are needed [189].

### 5.2. Vitamins Based Animal and Cellular Studies in Alzheimer’s Disease

However, in a streptozotocin (STZ)-induced AD mouse model, beta-carotene, a form of VitA, significantly improved measures of cognitive function and oxidative stress and reduced levels of toxic B-amyloid fragments [194]. In vitro and in vivo animal studies show that VitA and beta-carotene inhibit the oligomerization and aggregation of Aβ (Figure 3) [146,195,196]. Furthermore, VitC attenuated the advancement of neurodegeneration and improved behavioral deficits in a mouse model of AD [197]. Thus, a combination of VitA, VitC, and VitE therapy may provide a synergistic effect and act as adjuvants in preventing the progression of neurodegeneration in AD [198]. In an AD mouse model, neuronal dysfunction was significantly attenuated by intranasal administration of 9-cis retinoic acid (9-cis RA). This might be a novel therapeutic option for the preventive treatment of AD. The astrocyte activation, neuroinflammation, and Aβ aggregation were considerably reduced by 9-cis RA in the AD transgenic mouse model compared to controls (Figure 3). As a result, synaptic deficits were restored in the AD model compared to vehicle-treated mice [199]. Retinoids show strong therapeutic efficacy by preventing progressive neurodegeneration by inhibiting the neuroinflammatory processes in AD [200].

Although AD accounts for 60–80% of dementia, there are other types, such as vascular dementia (VD), frontotemporal dementia (FTD), and Wernicke-Korsakoff syndrome, etc. [201,202]. Interestingly, Wernicke-Korsakoff is a type of dementia caused by a lack of VitB1 (Thiamine), and VitB1 supplementation has been used to treat this type of dementia [203]. Similarly, symptoms of frontotemporal lobe degeneration appear in vascular dementia, and VitB1 supplementation significantly improves these symptoms [204]. VitB12 and folate (VitB9) deficiencies have also been seen in VD and AD patients [205]. Severe lack of niacin (VitB3) leads to pellagra, a disease characterized by diarrhea, dermatitis, and dementia, which is treated by niacin supplementation [159,206]. Thus, VitB shows therapeutic activity in AD and other dementias. In another study, pre-treatment with VitC prevented cognitive impairment and improved biochemical measures such as lowering proinflammatory cytokines, modulating anti-apoptotic activity, and contributing to the phosphorylation of p38 mitogen-activated protein kinase (MAPK) activation in the hippocampus of LPS-treated mice [207]. In an AlCl_3_ (100 mg/kg) induced rat model of Alzheimer’s disease (AD), ascorbic acid (100 mg/kg daily for 15 days) alleviated the biochemical and behavioral deficits along with neuropathological alteration by its antioxidative activity. Ascorbic acid shows AChE inhibitory and anti-proteolytic activity. Thus, the progression of AD is inhibited by ascorbic acid supplementation [208]. Thus, VitC improved cognition by modulating oxidative stress and neuroinflammatory parameters.

A study indicated that a low dose of VitC (200 and 400 mg/kg BW) confers protection from colchicine-induced, neuroinflammation-mediated neurodegeneration and cognitive impairments by scavenging free radicals. However, on the other hand, a higher dose of VitC (600 mg/kg BW) was responsible for the generation of oxidative stress, neuroinflammation, and cognitive impairment. Therefore, VitC exhibited dual activity with protection at lower doses and damage at higher [209].

VitD3 improved cognitive function by inhibiting neuroinflammatory and oxidative stress responses and improved cholinergic function in the mouse model of STZ-induced AD [210]. VitD improved the age-related cognitive decline in a rat model by modulating the activity of proinflammatory cytokines. VitD also decreased the amyloid burden responsible for cognitive impairment [211]. A recent study demonstrated that maxacalcitol, an analog of the active form of VitD, improved cognitive function and inhibited neuroinflammation induced by LPS in a rat model of AD, through Keap1/Nrf2 and MAPK-38p/ERK signaling pathways [212]. Thus, VitD and its analogs effectively prevent AD pathology in animal models and AD patients [177,183]. The deficiency of VitD promotes AD-like pathology by reducing the antioxidative potential. Enhanced production of amyloid-beta, the elevated phosphorylation status of tau, enhanced inflammatory loads, and reflected VitD deficiency in mice. A reduced concentration of the active form of VitD might improve the AD and dementia risk in a significant way [213]. In the D-Galactose-induced memory impairment mice model, neuroprotective activity is shown by VitD via SIRT1/Nrf-2/NF-kB signaling pathways [214]. Therefore, the VitD level should be checked and managed by the patients during AD treatment [215].

Extended administration of VitE provided better results as it counteracted effectively with Aβ plaques and NFT. Further, a type of VitE, α-tocopherol, prevented progressive neurodegeneration in animal models of AD [190]. In addition, α-tocopherol showed synergistic effects with antioxidative and anti-inflammatory compounds that are beneficial in treating AD [190,216].

In a retrospective study, a multivitamin exhibits its therapeutic potential to improve cognitive impairment. In this multivitamin retrospective study, higher folate concentration improved cognitive performance, as shown by its MMSE score. Folate deficiency is directly linked with hyperhomocysteinemia and is associated with worse cognitive performance. Further study will be needed to define the optimal vitamin status among affected individuals [191]. Another multivitamin-based study suggests a neuroprotective role of vitamin B6, B12, folate, and choline in hypoxia. This multivitamin treatment significantly alleviated hypoxia-induced memory deficits. Reduced tau hyperphosphorylation and decreased levels of homocysteine are detected by vitamin B6, B12, folate, and choline treatment. Memory functions were improved by this multivitamin approach [217]. Therefore, instead of a single vitamin-based approach, the multivitamin strategy might offer better therapeutic options for AD and MCI. Vitamin K2 prevents the Aβ induced neurotoxicity by the phosphatidylinositol 3-kinase (PI3K) associated signaling pathway [218].

In conclusion, vitamins have been studied for the treatment of AD and other types of dementia, and many of these studies have indicated vitamins’ beneficial role with evidence of VitA inhibiting the formation of Aβ plaques, and VitB, VitC, VitD, and VitE intervening with the progression of neurocognitive decline. As such, the role of many vitamins in adjuvant therapy treatments for AD has been underestimated [146]. Therefore, future clinical studies that employ vitamins in AD therapeutics might illuminate important details of how vitamins help alleviate AD symptoms.

## 6. Vitamins in Huntington’s Disease

**Figure 4:**
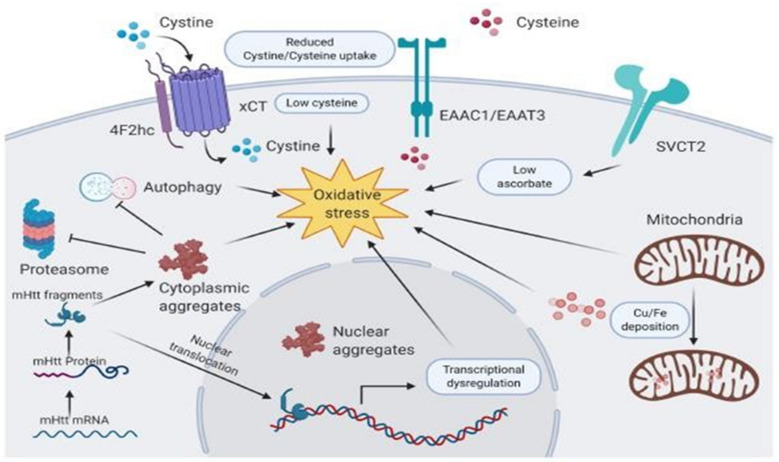
Etiology of oxidative stress behind Huntington’s disease (HD). In HD, reduced levels of ascorbate, cysteine, and antioxidants are observed. Excitatory amino acid transporter 3 (EAAT3/EAAC1) is the cysteine transporter, which is dysregulated and responsible for the reduced uptake of cysteine. Uptake of the oxidized form of cysteine is also reduced, which is mediated through the xCT and 4F2hc complex transporter. Sodium-dependent vitamin C transporters 2 (SVCT2)-mediated uptake of ascorbate (vitamin C), which is also reduced in HD, is responsible for the compromised antioxidant defense system in neurons. Cellular components are damaged because of metal deposition such as iron (Fe) and copper (Cu) in both cytoplasms and mitochondria, generating free radicals. Additional oxidative stress generates the mutant form of huntingtin aggregates (mHtt) in both the cytoplasm and nucleus. mHtt aggregates affect vital cellular processes such as mitochondrial dysfunction, proteostasis, and autophagy. In the nucleus, mHtt affects transcription factors involved in the cell’s antioxidant defense system. mHtt also affects DNA repair processes, which are responsible for damage and the error-prone repair system. Ascorbate (vitamin C) supplementation in HD improves the antioxidant defense system and prevents disease progression.

Huntington’s disease (HD) is also an example of a progressive, neurodegenerative disease of the CNS. Similar to PD and AD, motor abnormalities, dementia, and psychiatric problems are the main characteristics of HD [219,220]. HD is caused by a dominantly inherited genetic mutation that elongates a section of the huntingtin gene by the repetition of trinucleotide (CAG) segments (more than 36 repeats), leading to toxic intracellular polyglutamine aggregates and neuronal degradation [221,222] (Figure 4). Alterations in vitamins are involved in the pathogenesis of HD [223]. Oxidative stress is one of the major causative factors responsible for progressive neurodegeneration. Antioxidative therapy targeting the reactive oxygen species shows the potential impact of reducing the stress burden in associated neuronal cells [224]. Similar to AD and PD, vitamins also offer strong antioxidative activity and prevent progressive neurodegeneration in HD [225].

RA is the most studied form of VitA, and its receptors are present in the CNS [226]. RA effectively regulates adult brain physiology through its receptors [227,228,229]. However, the mechanism of action is not clearly understood. One of the main locations of RA receptors is in the striatum, a brain region critical for planning and the execution of movement, cognition, reward, and motivation [230,231]. One isoform of the RA receptor, known as retinoic acid receptor β (RARβ), is widely studied in PD, AD, and HD [232]. The characterization of RARβ transcriptional targets through genome-wide analysis identified possible mechanisms of action for RARβ activity [230]. RARβ controls the striatal pathways through transcription, energy metabolism, and neurotransmission through G-protein, c-AMP, and calcium signaling [230]. In HD transgenic mouse models, striatal signaling genes induced by retinoids are downregulated [233]. In addition, the RARβ receptor is sequestered inside huntingtin protein aggregates in the R6/2 mouse striatum [230]. These studies indicate that RA signaling is compromised in HD and contributes to HD pathology. Therefore, drugs that target this signaling pathway may offer a potential treatment for HD. Similar to AD and PD, more studies will be needed to reach any definite conclusion regarding the mechanism of action behind RA and its receptor in HD therapy [230].

The direct association of thiamine (VitB1) with HD is far from clear, but results have demonstrated that a deficiency in thiamine causes oxidative stress and neuroinflammation, which could contribute to the progression of HD [234,235,236]. Researchers investigated the role of supplemented thiamine in HD pathogenesis by assessing the viability of human B lymphocytes with and without the abnormal huntingtin gene [237]. Results indicated that energy metabolism is a vital step in HD pathogenesis, and thiamine deficiency caused a reduction in the cellular energy metabolism by altering the expression of various genes. In the HD human B lymphocyte model, the genes affected were glyceraldehyde-3-phosphate dehydrogenase (GAPDH), isocitrate dehydrogenase gene (IDH1), and the solute carrier family 19, member 3 (SLC19A3) gene. GAPDH, IDH1, and SLC19A3 are involved in the synthetic process of ATP and other high-energy-containing molecules [237]. Thus, thiamine controls the expression of genes involved in energy metabolism and could provide effective therapy for HD.

Recent results demonstrated that nicotinamide (VitB3) improved motor functioning and prevented the progression of 3-nitropropionic acid (NPA)-induced HD-associated neurodegeneration by balancing the redox state in a rat model. In addition, nicotinamide also improved histopathological parameters by decreasing the expression of lactate dehydrogenase, a marker of tissue degradation [238]. Thus, nicotinamide exhibits potent neuroprotective activity in a chemical-induced rat model of HD.

VitC, the most prescribed vitamin by clinicians, is involved in many body functions, including postural stability and bone health. Postural stability is the most common motor abnormality observed during neurodegenerative diseases, and bone density is significantly related to serum VitC concentration [239,240,241,242]. Therefore, VitC activity in HD has been widely explored. HD transgenic mouse models display dysregulation of ascorbate (VitC) within the cortical and striatal pathways [243]. These pathways regulate the activity of ascorbate with the help of the neurotransmitter glutamate in HD pathogenesis. Glutamate transporters on astrocytes are responsible for the removal of extracellular glutamate [244]. Both ascorbate release and glutamate uptake were impaired in a transgenic model of HD [245]. External ascorbate was responsible for increased glutamate uptake and, consequently, the inhibition of HD progression [245]. In addition, results indicated that for the uptake of glutamate, the release of ascorbate is needed, and in the absence of ascorbate, glutamate receptor activity is compromised by overactivation [245]. Overactive glutamate receptors were responsible for dysregulation of the corticostriatal pathway and contributed to HD pathogenesis in the R6/2 mouse HD model [246]. Furthermore, ascorbate deficiency contributed to behavioral deficits in HD by influencing the striatal pathway in the R6/2mice model, and intake of ascorbate alleviated behavioral abnormalities [245,246]. Further studies are needed to clarify the interactions between ascorbate and glutamate within the different signaling pathways and their influence on motor function.

Calcitriol (VitD) also plays an important role in muscle strength and bone density. As such, VitD deficiency is directly related to motor abnormalities [247,248]. A study of institutionalized HD patients found a high prevalence of VitD deficiency or insufficiency, and a positive association between calcifediol 25 (OH)D levels, an indicator of Vit D status, and ambulatory abilities was observed [221]. In a transgenic mouse HD model, supplementation of calcitriol significantly improved clinical symptoms and augmented the life span [249]. Thus, serum calcitriol is positively related to the treatment of HD.

In a localized and limited clinical trial, α-tocopherol, a major constituent of VitE, slowed the progression of motor abnormalities associated with HD [250]. Kasparová et al. studied the synergistic effects of CoQ10 and VitE in the NPA-induced rat model of HD. NPA-induced rat models exhibit reduced energy homeostasis. They found that creatine kinase (CK), an energy biomarker in brain diseases, was increased in their HD model. On the other hand, CoQ10, ATP, and the activity of the electron transport chain were reduced. Interestingly, supplementation of CoQ10 and VitE reversed these abnormalities [251].

In summary, for the treatment of HD, vitamins offer adjuvant therapeutic options. Both VitC and VitD prevent the progression of postural instability for HD patients. CoQ10, VitE, nicotinamide (VitB3), and VitB1 all show significant neuroprotective activity to minimize the load for HD patients. In addition, VitA and several intracellular mediators such as calcium and cyclic adenosine monophosphate are involved in the vitamin-mediated protection of HD. Large-scale clinical studies may be helpful to uncover the detailed mechanisms of action behind the neuroprotective effects of vitamins in HD pathogenesis.

## 7. Vitamins in Multiple Sclerosis

Multiple sclerosis (MS) is characterized by progressive neuroinflammation and subsequent neurodegeneration [252]. In the CNS, the body’s immune system attacks the myelin sheaths that coat and protect nerve fibers [253,254]. Movement abnormalities, vision problems, fatigue, and pain are common symptoms associated with MS. The etiology of MS is not clearly defined [255]. However, it is thought that both environmental and genetic factors are equally responsible for MS [256]. While there is no cure for MS, the usefulness of vitamins in MS therapeutics has been explored due to their antioxidant activities.

VitD has protective activity in MS that usually depends on the patient’s developmental stage. As such, VitD supplementation was more efficacious at ameliorating MS-like neuroinflammation in a juvenile/adolescent rat model of MS compared to adult aged animals [257]. Observational studies and clinical trials have shown that a reduced level of VitD in the blood is a risk factor for developing MS [258,259,260]. Supplementation with VitD shows potent anti-inflammatory activity by increasing the oxidation of white matter. However, overdosing with VitD can lead to hypercalcaemia, a toxic build-up of calcium in the blood [259,261,262,263,264]. In MS patients, supplementation with VitD enhanced blood perfusion, which supports tissue oxygenation and, therefore, reduced neuroinflammation and neurodegeneration [265]. Several studies suggested that the level of apolipoprotein E and two isoforms of VitD binding protein (DBP) in the cerebrospinal fluid could be utilized as potential biomarkers for the diagnosis of MS [266,267]. In contrast, another study found no association between DBP, MS, and VitD in blood samples from MS patients [268]. Therefore, larger controlled clinical trials are needed to evaluate the efficacy of VitD supplementation in MS therapies.

There is significant evidence from in vitro and in vivo studies of the beneficial effects of VitA/retinoic acid in treating MS [269,270]. VitA exhibited anti-inflammatory and antioxidative activity in the brain, and VitA serum levels were reduced in MS patients [269,271,272,273]. VitA improved the function of astrocytes, led to remyelination, and suppressed immune function in MS patients [218,269,274,275,276,277]. In contrast, results from one study demonstrated no correlation between serum VitA concentration and the progression of MS [278].

To date, few studies have investigated the potential therapeutic effects of the antioxidative ability of VitC or VitE in treating MS [279]. However, several studies have shown that as compared to a healthy individual, MS patients exhibit reduced levels of VitC [280,281,282]. Direct intrahippocampal injections of VitC in a rat model of MS improved memory dysfunction on passive avoidance learning [283]. Studies investigating VitE showed the improved function of oligodendrocytes and inhibition of factors related to the necrosis process in MS [284]. Due to the limited amount of information currently available for VitC and VitE in MS [285].

The role of VitB in MS is very controversial. Some studies have found that VitB levels are reduced in MS patients, while others show no relation between VitB levels and MS [286]. However, VitB may still be useful in MS therapies. Vitamins B9 and B12 regulate the immune function in MS by effectively improving the uptake of homocysteine, which is responsible for the synthesis of myelin sheaths [287,288,289,290,291]. In addition, the roles of VitB1, VitB3, and VitB6 in MS have been explored. VitB3 showed a remyelination ability and may be effective in the treatment of MS [292]. High-dose thiamine (VitB1) therapy improved the fatigue commonly associated with MS [293]. More clinical trials are needed to validate the role of VitB in MS therapies.

Because many observational studies have related VitD levels to MS risk, and because of the anti-inflammatory activities of VitD, there has been much focus on the utilization of VitD for the prevention and/or intervention of MS (for a comprehensive review, see Sintzel 2018) [260]. In addition, other vitamins such as VitA and VitE, due to their anti-inflammatory and antioxidative effects, may be helpful for adjuvants in MS treatments [269,270,294,295]. The role of VitB in MS is still controversial. Future clinical trials are necessary to determine the role of each vitamin in the potential treatment of MS.

## 8. Vitamins in Amyotrophic Lateral Sclerosis

Amyotrophic lateral sclerosis (ALS) is a fatal form of motor neuron disease (MND) characterized by progressive degeneration of motor neurons in the brain and spinal cord. The loss of motor neurons leads to the deterioration of whole-body muscle mass [296,297,298]. Similar to MS, very few studies have been performed regarding the role of vitamins in ALS. However, as in MS, there is a strong correlation between ALS and VitD supplementation. The active form of VitD is reduced in ALS patients and animal models of ALS [81,299]. VitD was shown to be protective in motor neurons in vitro, and plasma levels of VitD were directly correlated to the severity of the disease in ALS patients [300]. Genetic studies have shown that VitD is linked to ALS pathology through the regulation of various immune components such as toll-like receptors, major histocompatibility complex (MHC) class II molecules, poly (ADP-ribose) polymerase 1 (PARP1), and heme oxygenase-1 (HO-1) [81]. In addition, VitD influences ALS pathology through cell-signaling mechanisms, including Wnt/β-catenin, mitogen-activated protein kinase (MAPK), glutamate, prostaglandins, reactive oxygen species (ROS), matrix metalloproteinases, and nitric oxidase synthase [81]. In transgenic mouse models of ALS, VitD supplementation reduced symptoms of muscle weakness and improved motor functional capacity but did not prevent the final disease outcome [201,202,203,204,205,206,207,208,209,210,211,212,213,214,215,216,217,218,219,220,221,222,223,224,225,226,227,228,229,230,231,232,233,234,235,236,237,238,239,240,241,242,243,244,245,246,247,248,249,250,251,252,253,254,255,256,257,258,259,260,261,262,263,264,265,266,267,268,269,270,271,272,273,274,275,276,277,278,279,280,281,282,283,284,285,286,287,288,289,290,291,292,293,294,295,296,297,298,299,300,301,302,303]. In contrast, a recent study of ALS patients found no reduction in VitD levels, and no benefit for VitD supplementation for improving the prognosis of this disease [304]. Therefore, future studies are needed to determine the role of VitD in ALS pathophysiology.

The impact of VitE supplementation has been monitored in a clinical study. Results suggested that individuals who did not take the regular dose of VitE exhibited early death as compared to those who regularly took VitE supplementation. This study also concluded that VitE significantly improved motor functioning in ALS patients [305]. In a study focusing on transcriptional profiles, VitE prevented the death of NSC-34 motor neurons in ALS through the downregulation of the c-Jun N-terminal kinase (JNK) and p38 MAPK pathway (cell death), and upregulation of the extracellular signal-regulated kinase (ERK) pathways (cell survival) [306].

In summary, ALS is responsible for progressive muscular degeneration. VitD and Vit E may have beneficial activities in ALS by protecting motor neurons and improving motor symptoms. However, more studies are needed to understand the roles of these vitamins fully and to uncover the potential use of other vitamins in the treatment of ALS.

## 9. Vitamins in Prion Disease

Prion disease (PRD), also known as transmissible spongiform encephalopathies, is a rare progressive neurodegenerative disorder caused by abnormal prion protein accumulation, which leads to subsequent brain damage [307]. Structural differences occur between two forms of a prion protein [308]. PrPC is the normal prion protein, while the misfolded PrPSc (scrapie) is the pathogenic form [309]. The difference between the two versions of the prion protein is in the secondary structure [310,311]. Secondary structure α-helix motifs within the normal protein are converted to β-sheet secondary structures, which leads to protein misfolding and toxicity. The molecular mechanisms for this conversion are unclear [312]. Vitamins prevent the transformation of the normal form of the prion protein towards the pathogenic form [313]. Various micronutrients, including copper (Cu) and iron (Fe), are also involved in the vitamin-meditated maintenance of the normal form of the prion protein [314]. In addition, it is thought that oxidative stress and inflammation lead to the formation of the pathogenic form of the prion protein. Therefore, compounds or vitamins with antioxidative and anti-inflammatory characteristics should be beneficial in decreasing the risk of PRD [313].

Cobalamin (Cbl, Vitamin B12) deficiency plays a major role in the disturbance of the connection between the CNS and the peripheral nervous system (PNS) [315,316]. Cbl deficiency mainly affects glial cells, myelin sheaths, and the interstitium of the nervous system [317,318]. In the rat CNS, Cbl deficiency causes a reduction in the epidermal growth factor (EGF) and an enhancement of the activity of tumor necrosis factor-α (TNF-α), which leads to myelin damage and glial activation in both the CNS and PNS [319]. Interestingly, Cbl inhibits the nuclear localization of the NF-κB pathway, which is responsible for the upregulation of cytokines such as TNF-α and affects the conversion of the normal form of PrPC to the diseased form [320]. Another vitamin relevant to PRD is VitD2, which effectively crossed the BBB and suppressed PrPc oligomerization, a required step before PrPSc formation [321].

Metal homeostasis plays an important role in the maintenance of CNS [322]. Metals participate as cofactors in the vitamin-mediated therapeutic processes. Thus, the efficiency of vitamins is reduced in the absence of ample amounts of metal ions. Metals also participate in various enzyme-mediated activities in the CNS. Abnormalities in metal homeostasis might be responsible for the formation of ROS, which could contribute to the progression of neurodegenerative disease. PRD is also regulated by metal ion concentrations [323]. Harmful metals from animal proteins might induce the risk of PRD. The Mediterranean diet may have a protective role in preventing PRD [324]. The foundation of the Mediterranean diet is vegetables, fruits, herbs, various nuts, beans, and whole grains, which are rich in polyphenols that can cross the BBB and act on multiple targets to inhibit the formation of the toxic form of prion proteins [313]. Polyphenols are responsible for regulating harmful metal ions and inhibit the aggregated form of the prion protein [323]. Thus, the Mediterranean diet controls the activity of harmful metals and proves its efficacy in preventing PRD progression [323].

## 10. Vitamins in Age-Related Macular Degeneration

Not only influential in PD, AD, and HD, vitamins also exert their therapeutic response against age-related macular degeneration (AMD). Oral supplementation and modifications in diet show significant protection against AMD [325]. The Age-Related Eye Disease Study 2 (AREDS2) research group suggested that lutein/zeaxanthin plays a very vital role in the protection against AMD [326]. In AMD, the cone cell abnormalities caused by oxidative stress were significantly improved by VitD supplementation [327]. A clinical study on the Korean population exhibits that the degeneration of AMD progresses due to a lower level of VitD [328]. In the pathogenesis of AMD, the VitD metabolism plays a very novel role as suggested by a system-biology-based analysis. Therefore, we can say that vitamins are also very effective against AMD. More study will be needed to confirm the applicability and suitability of vitamins in AMD.

## 11. Conclusions and Future Prospective

Oxidative stress and neuroinflammation are the two major factors involved in the progression of neurodegenerative diseases. As such, compounds having antioxidative and anti-inflammatory properties should provide significant neuroprotection. In recent years, accumulating evidence has suggested a beneficial role for vitamins in protecting CNS diseases, as both WSV and FSV can protect neurons from death [329,330]. PD, AD, HD, MS, ALS, and PRD are the major neurodegenerative diseases found in humans. Animal models for these diseases have been utilized to prove the therapeutic efficacy of vitamins. However, the utilization of vitamins in therapies has been delayed due to studies which suggest no correlation between vitamin levels and neurodegenerative diseases. Despite this, several clinical trials support a potential role for vitamins in future therapies of neurodegenerative diseases. Hormesis is an important factor and should be taken into consideration while designing the dose of vitamins. Hormesis differentiates between the beneficial and toxic activity of vitamins at a particular dose. Multivitamin supplementation shows therapeutic solid potential for neurodegenerative diseases as compared to a single vitamin-based option. Multiple signaling pathways are involved in the multivitamin approach that can boost the antioxidative response manifold.

Here, we discussed both beneficial and controversial studies involving the role of vitamins in neurodegenerative diseases. Future studies involving different drug-dose paradigms, diverse animal models, and various geographical locations are necessary to determine precisely the potential roles for vitamins in therapies to treat neurodegenerative diseases. Moreover, human clinical trials are desirable to prove the efficacy and the protective role of vitamins in neurodegenerative diseases.

## Data Availability

Each of the authors confirms that this manuscript has not been previously published and is not currently under consideration by any other journal. Additionally, all of the authors have approved the contents of this paper and have agreed to submission policies.
